# Spinal apolipoprotein E is involved in inflammatory pain via regulating lipid metabolism and glial activation in the spinal dorsal horn

**DOI:** 10.1186/s13062-023-00444-z

**Published:** 2023-12-09

**Authors:** Siyi Liu, Shuting Yang, Xuan Zhu, Xiang Li, Xi Zhang, Xiaoqiong Zhou, Hong Cheng, Fu-Quan Huo, Qingxiang Mao, Lingli Liang

**Affiliations:** 1 Department of Physiology and Pathophysiology, School of Basic Medical Sciences, Xi’an Jiaotong University Health Science Center, Xi’an, Shaanxi People’s Republic of China; 2 Institute of Neuroscience, Translational Medicine Institute, Xi’an Jiaotong University Health Science Center, Xi’an, Shaanxi People’s Republic of China; 3 Department of Anesthesiology, Daping Hospital, Army Medical University, Chongqing, People’s Republic of China; 4 Department of Anesthesiology, Central Hospital Affiliated to Shandong First Medical University, Jinan, Shandong People’s Republic of China; 5 Key Laboratory of Environment and Genes Related to Diseases, Xi’an Jiaotong University, Ministry of Education, Xi’an, Shaanxi People’s Republic of China

**Keywords:** Apolipoprotein E, Astrocyte, Spinal dorsal horn, Complete Freund's adjuvant, Inflammatory pain

## Abstract

**Introduction:**

Inflammation and nerve injury promote astrocyte activation, which regulates the development and resolution of pain, in the spinal dorsal horn. APOE regulates lipid metabolism and is predominantly expressed in the astrocytes. However, the effect of astrocytic APOE and lipid metabolism on spinal cellular function is unclear. This study aimed to investigate the effect of spinal Apoe on spinal cellular functions using the complete Freund's adjuvant (CFA)-induced inflammatory pain mouse model.

**Methods:**

After intraplantar injection of CFA, we assessed pain behaviors in C57BL6 and *Apoe* knockout (*Apoe*^*−/−*^) mice using von Frey and Hargreaves’ tests and analyzed dorsal horn samples (L4-5) using western blotting, immunofluorescence, quantitative real-time polymerase chain reaction, and RNA sequencing.

**Results:**

The Apoe levels were markedly upregulated at 2 h and on days 1 and 3 post-CFA treatment. Apoe was exclusively expressed in the astrocytes. *Apoe*^*−/−*^ mice exhibited decreased pain on day 1, but not at 2 h, post-CFA treatment. *Apoe*^*−/−*^ mice also showed decreased spinal neuron excitability and paw edema on day 1 post-CFA treatment. Global transcriptomic analysis of the dorsal horn on day 1 post-CFA treatment revealed that the differentially expressed mRNAs in *Apoe*^*−/−*^ mice were associated with lipid metabolism and the immune system. Astrocyte activation was impaired in *Apoe*^*−/−*^ mice on day 1 post-CFA treatment. The intrathecal injection of *Apoe* antisense oligonucleotide mitigated CFA-induced pain hypersensitivity.

**Conclusions:**

Apoe deficiency altered lipid metabolism in astrocytes, exerting regulatory effects on immune response, astrocyte activation, and neuronal activity and consequently disrupting the maintenance of inflammatory pain after peripheral inflammation. Targeting APOE is a potential anti-nociception and anti-inflammatory strategy.

**Supplementary Information:**

The online version contains supplementary material available at 10.1186/s13062-023-00444-z.

## Introduction

Chronic pain resulting from injury and inflammation is a prevalent, persistent, and debilitating health concern [1]. Spinal glial cells, such as astrocytes and microglia are reported to regulate nociception under chronic pain conditions [2]. Astrocytes, which are a major glial cell type in the central nervous system (CNS), regulate neurotransmitter and calcium homeostasis, synapse formation, maturation, and elimination, blood–brain barrier function, and ion homeostasis, providing nutritional and trophic support to the brain [3, 4]. In particular, astrocytes exhibit enhanced lipid metabolism-related enzyme activity and are the major source of cholesterol and phospholipids in the CNS [5, 6]. Under pathological conditions, such as Alzheimer’s disease (AD) and chronic pain, astrocytes are reactive and exhibit distinct metabolic features when compared with the resting astrocytes [7]. Apolipoprotein E4 (APOE4), which is the strongest genetic risk factor for late-onset AD in humans, is reported to impair lipid metabolism in human astrocytes and microglia. Additionally, APOE4 upregulates matrisome pathway, chemotaxis, glial activation, and lipid metabolism in astrocytes through astrocyte-neuron communication [8]. However, the role of astrocytic lipoprotein and lipid metabolism in the development of chronic pain is unclear.

APOE is a key regulator of lipid homeostasis and is primarily expressed in astrocytes in the brain [9]. Additionally, APOE plays a crucial role in the metabolic network between astrocytes and neurons, transporting essential nutrients and antioxidants to the neurons [9–11]. Furthermore, APOE is associated with neuroinflammation and synaptic plasticity in the nervous system [9, 12, 13]. Among three *APOE* alleles/variants: *APOE2*, *APOE3*, and *APOE4*, in humans, the *APOE4* allele is a genetic risk factor for developing AD, while *APOE* is also associated with chronic pain [14, 15]. The plasma levels of APOE are upregulated in patients with chronic low back pain [16]. In contrast to humans, mice harbor a single *Apoe* allele [5]. Animal studies have identified that Apoe in the spinal microglia and dorsal root ganglion (DRG) satellite glial cells mediates neuropathic pain [15, 17]. APOE is predominantly expressed and secreted by astrocytes in the spinal cord [12, 17, 18]. Thus, this study aimed to investigate the potential role of astrocytic Apoe in the pathological processes of chronic pain and its regulatory effects on spinal cellular function under chronic pain conditions.

This study investigated the role of spinal astrocytic Apoe in in inflammatory pain in response to intraplantar complete Freund's Adjuvant (CFA) injection. We observed astrocytic Apoe levels in the spinal dorsal horn after CFA treatment. We further compared nociceptive responses and neuronal activity of spinal nociceptive neurons in control mice and *Apoe*^*−/−*^ mice when subjected to CFA treatment. RNA sequencing and transcriptomic analysis, along with immunofluorescence revealed differential cellular changes in CFA-treated *Apoe*^*−/−*^ mice. Finally, we observed the effect of the intrathecal injection of *Apoe* antisense oligonucleotide (ASO) on CFA-induced inflammatory pain hypersensitivity. These findings indicate the unique role of spinal astrocytic APOE in inflammation-induced pain and enhance our understanding of lipid metabolism in astrocytes in chronic pain conditions.

## Materials and methods

### Animals

Male C57BL/6 J (C57) and *Apoe*^*−/−*^ mice (B6/JGpt-Apoeem1Cd82/Gpt, Cas9-knockout, C57BL/6JGpt, T001458) aged 7–8 weeks with a bodyweight of 20–25 g were obtained from Gem-pharmatech Co., Ltd (Nanjing, China). All mice were housed in the animal facility of Xi`an Jiaotong University under the following conditions: temperature, 22–25 °C; circadian cycle, 12-h light–dark cycle. Animal experiments were approved by the Institutional Animal Ethics Committee of Xi’an Jiaotong University Health Science Center (No. 2018-334), ensuring compliance with legal and ethical guidelines. Strict guidelines have been adhered to minimize the use of mice and reduce animal suffering during the experiment.

### Inflammatory pain model establishment

The CFA-induced inflammatory pain model was established by administering CFA into the left hind paw plantar of mice. An equal volume of CFA (Cat#: 344289; Sigma-Aldrich, St. Louis, MO, USA) and physiological saline were mixed to generate an oil-saline (1:1) emulsion for administration. Each mouse in the CFA group was intraplantarly administered with 10 μL of the prepared emulsion, while that in the control group was intraplantarly administered with 10 μL of physiological saline.

To establish the formalin-induced inflammatory pain model, a 5% formalin solution was prepared using a 10% neutral buffered formalin solution (Cat#: HT501128; Sigma-Aldrich) in physiological saline (1:1 ratio). The 5% formalin solution was subcutaneously injected into the left paw plantar. The control mice were injected with NS. The nociceptive behaviors, including paw withdrawal, licking, and paw flinching, were recorded at different time points after formalin administration.

### Behavioral tests

The *von* Frey test was performed to quantify mechanical allodynia and mechanical hyperalgesia in mice using *von* Frey filaments weighing 0.16 g and 0.4 g, respectively [17, 19]. Mice were allowed to acclimatize to the environment for at least three days before behavioral experiments. Briefly, the unrestrained mice were transferred to a rectangular Plexiglass chamber placed on an elevated mesh platform after a 30-min adaptation period. Each filament was applied to the bottom of the hind paws 10 times. A positive response was recorded if the mouse exhibited sudden paw withdrawal, licking, or shaking. The percentage of paw withdrawal responses was calculated and expressed as paw withdrawal frequency (PWF, %).

Hargreaves’ test was used to assess the thermal hypersensitivity of mice with an Analgesic Meter (Model 37370, Ugo Basile, Gemonio, Italy) [17]. After acclimation for 30 min, the paw withdrawal latency (PWL) value was automatically recorded from the moment the heat source was activated until a strong paw withdrawal response was recorded. Each paw was tested five times at 5-min intervals. The average PWL was calculated for each paw in one trial. The cutoff time was set to 20 s to avoid tissue damage.

### Paw edema evaluation

The paw volume was measured using an IITC 520 plethysmometer (IITC Life Science Instruments, CA). Measurements were recorded at baseline (V_0_) and at different time points (V_I_) post-CFA administration. To measure the paw volume (in mL), the paw was placed into a water bath during measurement. The paw volume (mL) was displayed on the readout and was used to determine the degree of paw edema. Paw edema was assessed by calculating the percentage of increase in paw volume after inflammation as follows: paw edema = [(V_I_ − V_0_)/V_0_] × 100.

### Intrathecal injection of ASO via lumbar puncture

The sequences of *Apoe* ASO described in a recent study and negative control (NC) ASO were GGTGAATCTTTATTAAAC and CCTATAGGACTATCCAGGAA, respectively [17]. ASOs were synthesized by GenePharma (Shanghai, China) and dissolved in sterile 0.01 M phosphate-buffered saline (PBS; PH 7.4) immediately before administration. To inhibit RNase H-mediated degradation and promote cellular uptake, the ASOs were subjected to 2′-O-methoxyethyl modifications on the 5′-terminus and 3′-terminus and phosphorothioate modifications. The mice were intrathecally administered with ASO or PBS 3 days before CFA administration. The injection was performed at the L4–L5 posterior intervertebral space using a sterile insulin needle syringe under inhalational anesthesia via 3% isoflurane. To enhance the spread of the injected solution around the spinal cord and prevent efflux to the periphery, the needle was not moved for 30 s after the injection.

### Western blotting

The spinal dorsal horn from the L4–L5 spinal segment was collected after the mice were decapitated under isoflurane anesthetization. The tissues were homogenized in cold radioimmunoprecipitation assay lysis buffer (Cat#: P0013D; Beyotime, Shanghai, China) with phenylmethanesulfonyl fluoride (100 mM; Cat#: ST506; Beyotime) and protease inhibitor cocktail (100 × , Cat#: HY-K0010; MedChemExpress, Shanghai, China). The lysates were centrifuged at 1000 g and 4 °C for 15 min. The supernatant was used as the cytosolic fraction and subjected to western blotting. The protein concentration was determined using the bicinchoninic acid assay (Cat#: P0010; Beyotime). The samples were incubated at 99 °C for 5 min and loaded onto a 10% stacking/7.5% separating sodium dodecyl sulfate–polyacrylamide gel electrophoresis gel quick preparation kit (Cat#: P0012AC; Beyotime). The resolved proteins were transferred to a nitrocellulose membrane (pore size: 0.45 μm). The membrane was blocked with QuickBlock™ blocking buffer (Beyotime) and probed with rabbit polyclonal anti-Apoe antibodies (purified full-length native protein corresponding to mouse Apoe; 1:1000; Cat# ab20874; Abcam, Cambridge, MA, USA) or mouse anti-β-actin antibodies (1:2000; Cat#: sc-8432; Santa Cruz Biotechnology, Santa Cruz, CA, USA) at 4 °C overnight. After washing thrice with Tris-buffered saline with 0.05% Tween 20 (TBST), the membrane was incubated with peroxidase-conjugated goat anti-rabbit IgG (1:20,000; Cat#: A9169; EMD Millipore, Darmstadt, Germany) or goat anti-mouse IgG (H + L) (1:20,000; Cat#: DY60203; DEEYEE, Shanghai, China). Immunoreactive signals were developed using an enhanced chemiluminescence (ECL) kit (Clarity Western ECL Substrate; Cat#: WBKLS0500; EMD Millipore) and digitized into a digital film using a Champchemi System with SageCapture software (Sagecreation Service for Life Science, Beijing, China). The densitometric images were analyzed using ImageJ Software (Wayne Rasband, National Institutes of Health, Bethesda, MD, USA). The β-actin was used as an internal control. The average value in the saline-treated groups was set to 100% after the expression of Apoe was normalized to that of β-actin. The relative Apoe level was presented as a ratio of the normalized value to the average value of all saline-treated groups. 

### Immunofluorescence analysis

Mice were anesthetized and transcardially perfused with physiological saline, followed by perfusion with 4% paraformaldehyde (PFA) in 0.01 M PBS. The whole spinal cord was excised, post-fixed overnight in the same solution, and dehydrated using gradient sucrose solution (20% and 30%) until it sank at 4 °C. The L4–L5 spinal cord was dissected and sliced into sections with a thickness of 30 μm using a Leica CM1860 cryostat (Leica, Wetzlar, Germany). For single labeling, the sections were washed with 0.01 M PBS, preincubated with blocking buffer (0.01 M PBS supplemented with 0.3% Triton X-100 and 10% goat serum) for 1 h at 25 °C, and incubated with rabbit polyclonal anti-Apoe (1:500; Cat#: ab20874; Abcam), rabbit polyclonal anti-Fos (1:10,000; Cat#: ab190289; Abcam), rabbit IgG anti-p-Jun (Ser63; p-c-Jun) (1:200; Cat#: CY6576; Abways, Shanghai, China), or mouse monoclonal anti-glial fibrillary acidic protein (GFAP) antibodies (clone G-A-5, purified from hybridoma cell culture; 1:500; Cat#: G6171; Sigma-Aldrich) overnight at 4 °C. After washing thrice with PBS, the sections were incubated with donkey Alexa Fluor 488-conjugated anti-rabbit secondary antibodies (1:200; Cat#: ab150073; Abcam) for Apoe and Fos, Cy3-conjugated goat anti-rabbit secondary antibodies (1:500; Cat#: AP132C; EMD Millipore) for p-Jun, or Cy3-conjugated goat anti-mouse secondary antibodies (1:500; Cat#: AP124C; EMD Millipore) for GFAP at 4 °C for 2 h. For double labeling of Apoe and GFAP, the sections were incubated with the rabbit anti-Apoe antibody and mouse anti-GFAP antibodies, followed by incubation with a mixture of the corresponding secondary antibodies. The microscopic images were captured using an Axio Scope A1 fluorescence microscope (ZEISS, Carl Zeiss, Germany) and processed and quantified using ImageJ Software (Wayne Rasband). The Apoe-positive, GFAP-positive, or p-Jun-positive areas and the total area of the spinal dorsal horn were quantified and the percentage of positive area in the partial or whole spinal dorsal horn was calculated. The correlation between the colocalization of Apoe and GFAP was examined using Pearson’s correlation coefficient as described previously [17]. Briefly, the images of the double-labeled sections were split according to the channels using Fiji’s “Coloc 2” plugin in Image J Software. The fluorescence signals were measured after the segmentation of the background by thresholding. Pearson’s r-value will be shown in a PDF file after the channel 1 and 2 images were selected as red and green signals, respectively. A strong correlation between the two labeled signals was indicated when the r-value was greater than 0.5. For immunostaining, all images from at least 3 mice were randomly chosen. The minimal number of images from one mouse was 3.

### Quantitative real-time quantitative polymerase chain reaction (qRT-PCR)

The spinal dorsal horn from the L4–L5 spinal segment was excised after the rapid decapitation of mice under isoflurane anesthetization. Total RNA was extracted using the Trizol method (Cat#: R0026; Beyotime). The concentration of RNA was measured using ultraviolet spectrophotometry and the NanoDrop™ 2000 Spectrophotometer (Thermo Fisher Scientific, Waltham, MA, USA). The isolated RNA was reverse-transcribed into complementary DNA (cDNA) in triplicate in a 20-μL reaction mixture comprising 300 ng cDNA in 5 μL RNase-free H_2_O and 5 μL Hifair® II first strand cDNA synthesis SuperMix for qPCR (Cat#: 11120ES60; Yeasen, Shanghai, China) with a T100 PCR thermal cycler (Cat#: 1861096; Bio-Rad Hercules, CA, USA). qRT-PCR analysis was performed with 4 µL of template cDNA and 16 µL Hieff® qPCR SYBR Green Master Mix (Cat#: 11201ES03; Yeasen) using a BIO-RAD CFX96 real-time PCR system (Bio-Rad). The PCR conditions were as follows: 95 °C for 3 min, followed by 40 cycles of 95 °C for 10 s, 60 °C for 30 s, and 72 °C for 30 s. The primer sequences were as follows: *Apoe*, 5′-GCTGTGAAGGGGGAGAGAAC-3′ (forward) and 5′-ATTGGCCAGTCAGCTCCTTC-3′ (reverse); *Gapdh*, 5′-TCGGTGTGAACGGATTTGGC-3′ (forward) and 5′-TCCCATTCTCGGCCTTGACT-3′ (reverse). *Gapdh* was used as the internal control. The mRNA levels in the ipsilateral (injection side) and contralateral sides of the same animal were examined using the ΔCt method (2^−ΔCt^).

### RNA sequencing and bioinformatics analysis

At 24 h post-bilateral intraplantar injection of CFA, bilateral L4–L5 spinal dorsal horns were dissected from C57 or *Apoe*^*−/−*^ mice subjected to rapid decapitation under isoflurane anesthetization. The L4–L5 spinal dorsal horn from one mouse was treated as an independent biological replicate. In total, 8 samples (four independent biological replicates per group) were sent to BGI Genomics Inc. (Wuhan, China). RNA sequencing was performed as described previously [20]. Briefly, total RNA was extracted and purified according to the manufacturer’s instructions (BGI Genomics). RNA sequencing was outperformed using an Illumina Hiseq × 10 platform High Output Mode in a 50-bp paired-end configuration with at least 20 million (M) reads per sample (BGI Genomics). Eight samples were then subjected to multiplexing, sequencing, differential gene expression analysis, and transcript expression analysis. The gene expression levels between the groups were compared using Student’s *t*-test and transformed into log_2_ values. Differentially expressed genes (DEGs) were then extracted based on the criterion adjusted *P* < 0.05 and subjected to Gene Ontology (GO) and Kyoto Encyclopedia of Genes and Genomes (KEGG) pathway enrichment analyses using DAVID Bioinformatics Resources 6.7 with the phyper function in R software. Enrichment was considered significant at Q ≤ 0.05.

### Statistical analysis

All statistical analyses were performed using GraphPad Prism 9 (GraphPad, San Diego, CA, USA). Experimental animals were randomized and assigned to various treatment groups. For behavioral tests, 6–10 mice were included in each group. Meanwhile, four mice were included in each group for RNA sequencing analysis of bilateral spinal cord dorsal horn. Three independent qRT-PCR and western blotting experiments were performed for statistical analysis of different biological repeats. The correlation of double-label immunofluorescence analysis results was examined using Pearson’s correlation coefficient.

## Results

### CFA upregulated Apoe expression in the lumbar spinal dorsal horn

**Fig. 1:**
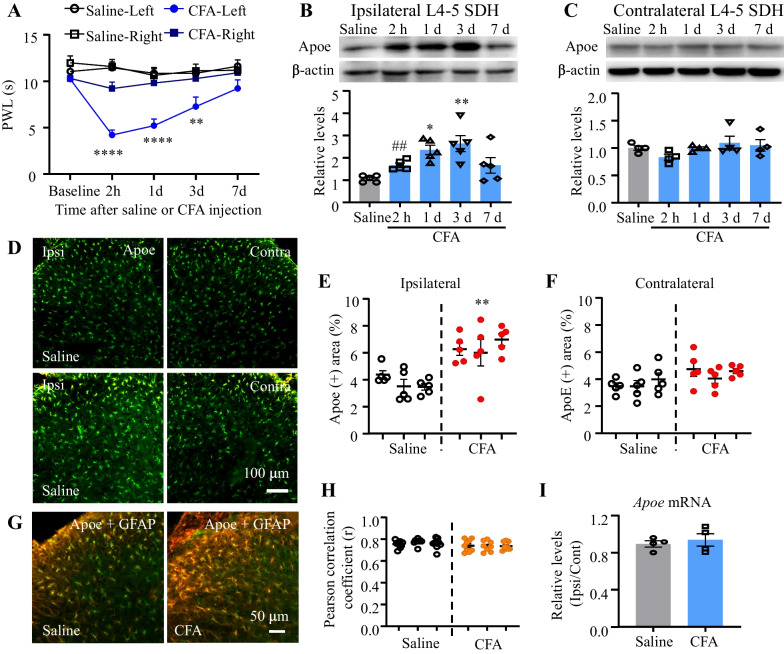
Complete Freund’s adjuvant (CFA) upregulates Apoe protein expression in the spinal dorsal horn. **A** Paw withdrawal latency (PWL) to thermal stimulation in mice after physiological saline or CFA treatment. N = 6–16 mice/group. **p < 0.01, ****p < 0.0001 versus the saline-treated mice by two-way analysis of variance (ANOVA) followed by post hoc Tukey test. **B–C** Representative blots and quantification of Apoe expression in the ipsilateral (**A**) and contralateral spinal dorsal horns (**B**) after saline or CFA treatment. N = 4–5 mice/group. ^##^*p* = 0.001 compared with saline-treated mice (two-tailed unpaired Student’s *t*-test). ^*^p = 0.0117, ^**^p = 0.0020 compared with saline-treated mice (one-way ANOVA, followed by Tukey’s *post-hoc* test). **D–F** Immunofluorescence staining (**C**) and quantification (**D**, **E**) of Apoe in the ipsilateral (Ipsi; **E**) and contralateral (Contra; **F**) spinal dorsal horns on day 1 post-saline or CFA treatment. N = 15 sections from 3 mice per group. The individual dot indicates one value from one section. All dots in one cluster present all values from one mouse. ^**^p = 0.003 compared with saline-treated mice (two-tailed unpaired Student’s *t*-test). Bar: 100 μm. **G–H** Colocalization of Apoe with GFAP and Pearson correlation coefficients (r) between Apoe staining and GFAP staining. N = 26 sections from 3 mice per group. Bar: 50 μm. **I** The mRNA level of *Apoe* in the spinal dorsal horn on day 1 post-saline or CFA treatment. *N* = 4 mice/group. Two-tailed unpaired Student’s *t*-test

The results of the Hargreaves test illustrated that CFA injection into the plantar surface of the mice’ left hind limb provoked thermal hyperalgesia, characterized by an obvious decrease in the PWL of the ipsilateral hind paw tested at 2 h and on day 1 and 3 after the injection, compared to the baseline control before CFA administration or saline-treated mice (Fig. 1A). Expectedly, the intraplantar saline injection resulted in no significant differences in PWL at the tested time points compared with the baseline control before saline injection (Fig. 1A). The results of western blotting demonstrated that Apoe expression was markedly upregulated (approximately 1.67-fold of) at 2 h post-CFA injection compared to the saline-treated mice (two-tailed unpaired Student's *t*-test) (Fig. 1B). The expression of Apoe protein continued to increase on days 1 (approximately 2.36-fold) and 3 (approximately 2.64-fold) post-CFA treatment but partially recovered on day 7 (approximately 1.66-fold) post-CFA treatment (Fig. 1B). Additionally, the Apoe-positive area in the contralateral spinal dorsal horn increased at all tested time points post-CFA treatment (Fig. 1C). The upregulated Apoe expression was further confirmed using immunofluorescence. Immunofluorescence analysis revealed that the Apoe-positive area in the ipsilateral spinal dorsal horn of CFA-treated mice was higher than that in the ipsilateral spinal dorsal horn of saline-treated mice on day 1 post-CFA treatment (Fig. 1D–F). Pearson’s correlation coefficient value for the double labeling of Apoe and GFAP was similar between saline-treated and CFA-treated mice (Fig. 1G, H), suggesting that astrocytes were the primary source of Apoe production at the early stage (day 1) of CFA-induced inflammatory pain. CFA did not affect the spinal dorsal horn mRNA levels of *Apoe* (Fig. 1I). However, the spinal Apoe protein level was upregulated in the early stages of CFA-induced inflammatory pain. This suggests that Apoe is involved in the development of inflammatory pain.

### The less pain hypersensitivity in *Apoe*^*−/−*^ mice in response to CFA injection

**Fig. 2:**
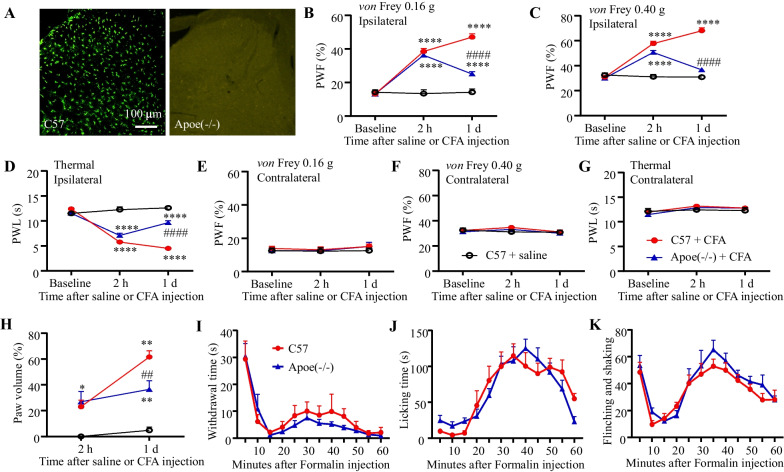
Complete Freund’s adjuvant (CFA) suppressed pain hypersensitivity and inflammation in *Apoe*^*−/−*^ mice. **A** The loss of Apoe immunofluorescence staining in *Apoe*^*−/−*^ mice. Bar: 100 μm. **B–G** Paw withdrawal frequency (PWF) in response to 0.16 g (**B**, **C**) and 0.4 g (**D**, **E**) *von* Frey filament stimulation and paw withdrawal latency (PWL) in response to thermal stimulation (**F**, **G**) on the ipsilateral (**B**, **D**, **F**) and contralateral (**C**, **E**, **G**) sides of C57BL6 (C57) or *Apoe*^*−/−*^ mice after physiological saline or CFA treatment. N = 6–16 mice/group. ^****^p < 0.0001 compared with saline-treated C57 mice; ^####^*p* < 0.0001 compared with CFA-treated C57 mice (two-way analysis of variance (ANOVA), followed by Tukey’s *post-hoc* test).** H** Paw edema evaluated based on the percentage of increased paw volume in C57 or *Apoe*^*−/−*^ mice after saline or CFA treatment. N = 6–8 mice/group. ^*^p = 0.0222 (2 h), ^****^p < 0.0001 (24 h) compared with saline-treated C57 mice; ^##^*p* = 0.006 compared with CFA-treated C57 mice (two-way ANOVA, followed by Tukey’s *post-hoc* test). **I–K** The duration of paw withdrawal (**H**) and licking (**I**) and the number of paw flinching and shaking events (**J**) in C57 and *Apoe*^*−/−*^ mice within 1 h post-intraplantar administration of 5% formalin. N = 8 mice/group. Two-way ANOVA

To determine the role of spinal Apoe in the development of inflammatory pain, CFA-induced pain hypersensitivity in *Apoe*^*−/−*^ and C57 mice was comparatively analyzed. The complete knockout of Apoe in mice was verified using immunofluorescence staining. *Apoe*^*−/−*^ mice did not exhibit Apoe immunostaining signals in the spinal cord (Fig. 2A). In C57 mice, the PWF in response to stimulation with 0.16 and 0.4 g *von* Frey filaments were higher (Fig. 2B, C) and PWLs to thermal stimulation were lower (Fig. 2D) on the ipsilateral sides in the CFA-treated group than those in the saline-treated group at 2 h and on day 1 post-CFA treatment. These data indicate that CFA treatment sufficiently induced mechanical and thermal pain hypersensitivity in C57 mice. However, *Apoe*^*−/−*^ mice exhibited decreased PWF (Fig. 2B, C) and prolonged PWL (Fig. 2E) when compared with C57 mice on day 1, but not at 2 h, post-CFA treatment. This indicates that *Apoe*^*−/−*^ mice did not exhibit persistent pain hypersensitivity. Thus, Apoe may be involved in the transition from acute pain to persistent pain after peripheral inflammation. Additionally, PWFs or PWLs on the contralateral sides were similar between the groups (Fig. 2E–G), indicating that Apoe deficiency did not affect basal nociceptive responses to either mechanical or thermal stimulation. Next, paw edema in CFA-treated C57 and *Apoe*^*−/−*^ mice was comparatively analyzed. Paw edema was evaluated by measuring the percentage increase in paw volume using an IITC 520 plethysmometer. In this study, paw edema was not observed in saline-treated mice (Fig. 2H). CFA treatment induced paw edema in C57 mice at 2 and 24 h post-treatment (Fig. 2H). Paw volume was similar between *Apoe*^*−/−*^ and C57 mice at 2 h post-CFA treatment. Similar to nociceptive responses, paw edema in *Apoe*^*−/−*^ mice was significantly alleviated when compared with that in C57 mice at 24 h post-CFA (Fig. 2H).

To further verify the effect of spinal *Apoe* knockout on acute inflammatory pain, formalin-induced acute pain in *Apoe*^*−/−*^ mice was examined. Paw withdrawal time (Fg. 2I), licking time (Fig. 2J), or flinching and shaking numbers (Fig. 2K) were not significantly different between *Apoe*^*−/−*^ and C57 mice.

### The less Fos expression in *Apoe*^*−/−*^ mice in response to CFA injection

**Fig. 3:**
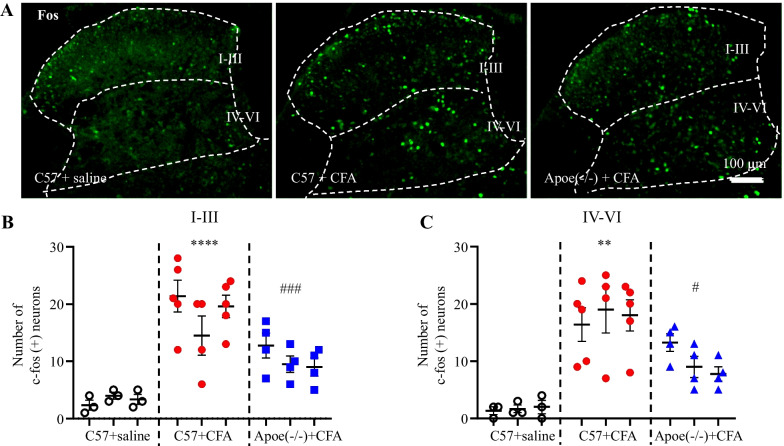
Complete Freund’s adjuvant (CFA) downregulates Fos expression in the spinal dorsal horn of *Apoe*^*−/−*^ mice. The left hind paw of all mice was repeatedly brushed for 1 min two hours before cardiac perfusion. **A** Immunofluorescence analysis of Fos expression in the three groups. **B–C** Quantification of Fos-positive neurons in the superficial dorsal horn (Layers I–III, b) and deep dorsal horn (Layers IV–VI, c). N = 3 mice/group (3–5 sections/mouse). ^****^p < 0.0001 compared with physiological saline-treated C57 mice; ^##^*p* = 0.0012, ^###^*p* = 0.0002 compared with CFA-treated C57 mice. Two-way analysis of variance, followed by Tukey’s *post-hoc* test

Next, this study investigated the effect of spinal Apoe on neuronal hyperexcitability by evaluating the Fos expression levels in the spinal dorsal horn of CFA-treated *Apoe*^*−/−*^ mice. On day 1 post-CFA treatment, the mouse paw was brushed repeatedly for 1 min to induce Fos expression in the spinal dorsal horn. In C57 mice, the number of Fos-positive neurons in both superficial (Layers I–III) and deep dorsal horns (Layers IV–VI) in the CFA-treated group was significantly higher than that in the saline-treated group (Fig. 3A–C). The number of Fos-positive neurons in CFA-treated *Apoe*^*−/−*^ mice was significantly lower than that in CFA-treated C57 mice (Fig. 3A–C). This indicates that spinal Apoe deficiency impaired noxious stimulus-induced spinal neuron excitability.

### Differentially expressed mRNAs between *Apoe*^*−/−*^ and C57 mice after CFA treatment

**Fig. 4:**
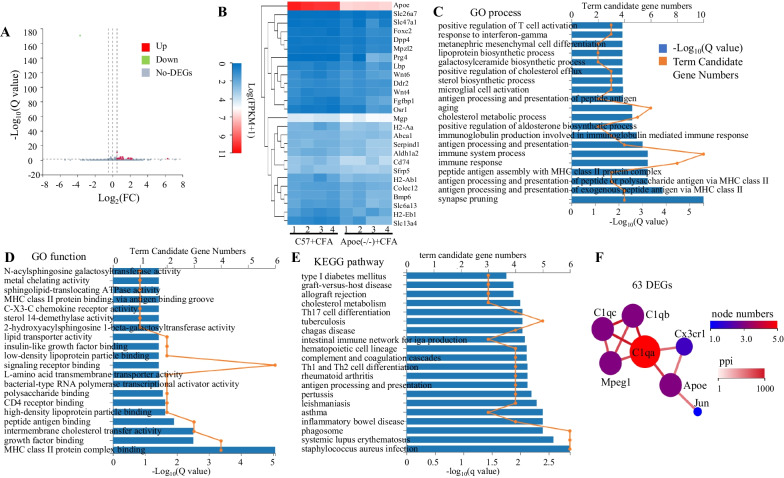
Differentially expressed genes (DEGs) between *Apoe*^*−/−*^ and C57BL6 (C57) mice after complete Freund’s adjuvant (CFA) treatment. **A** Volcano plot of 63 DEGs between CFA-treated C57 and *Apoe*^*−/−*^ mice. **B** Heatmap of 26 DEGs determined based on log_2_ (fold change (FC)) > 0.5 or <  − 0.5. **C** The enrichment of DEGs in the Gene Ontology (GO) term molecular function. **D** The enrichment of DEGs in the GO term biological process. **E** Kyoto Encyclopedia of Genes and Genomes (KEGG) pathway analysis of 63 DEGs. **F** Protein–protein interaction (PPI) network of 63 DEGs, including *Apoe*, *Jun*, *Cx3cr1*, and *C1qa*

To investigate the potential molecular mechanism through which Apoe mediates spinal neuron hyperexcitability and inflammatory pain, RNA sequencing was performed to identify differentially expressed mRNAs between CFA-treated C57 and *Apoe*^*−/−*^ mice. In total, 63 DEGs were identified between CFA-treated C57 and *Apoe*^*−/−*^ mice (Q < 0.05) (Fig. 4A, Additional file 1: Excel 1). Furthermore, hierarchical clustering analysis revealed 26 DEGs (log_2_-(fold change) > 0.5 or <  − 0.5) (Fig. 4B).

GO enrichment analysis revealed that the 63 DEGs were enriched in different level 2 GO terms as follows: biological process term: synapse pruning, antigen processing and presentation, immune response, cholesterol metabolic process, microglial cell activation, and positive regulation of T cell activation (Fig. 4C; Additional file 2: Excel 2); molecular function term: binding, transporter activity, transcription regulator activity, and catalytic activity (Fig. 4D; Additional file 3: Excel 3). In the level 2 GO term binding function, the DEGs could bind with major histocompatibility (MHC) class II protein complex, growth factors, peptide antigens, high-density lipoprotein particles, CD4 receptors, polysaccharides, signaling receptors, low-density lipoprotein particles, and insulin-like growth factor (Fig. 4D). Meanwhile, in the level 2 GO term transporter activity, the DEGs were involved in transmembrane cholesterol transfer activity, transmembrane transport of L-amino acids, and lipid transport activity (Fig. 4D).

KEGG pathway analysis revealed that the DEGs were enriched in the following level 2 KEGG pathways: immune system and immune disease (Fig. 4E; Additional file 4: Excel 4). In the level 2 KEGG pathway immune system, DEGs were associated with various pathways, such as antigen processing and presentation, Th1 and Th2 cell differentiation, complement and coagulation cascades, hematopoietic cell lineage, intestinal immune network for IgA production, and Th17 cell differentiation (Fig. 4E).

Protein–protein interaction (PPI) network of 63 DEGs revealed that Apoe was associated with Jun, Cx3cr1, and C1qa (Fig. 4F). Cx3cr1 is a receptor that is specifically expressed in microglia [21]. MAPK8, which is specifically expressed in spinal astrocytes and is required for the maintenance of bilateral mechanical allodynia induced by CFA, activates Jun [22]. These findings indicate that Apoe deficiency exerts antinociceptive effects by significantly modulating glial function under inflammatory pain conditions.

### The less astrocyte activation in *Apoe*^*−/−*^ mice in response to CFA injection

**Fig. 5:**
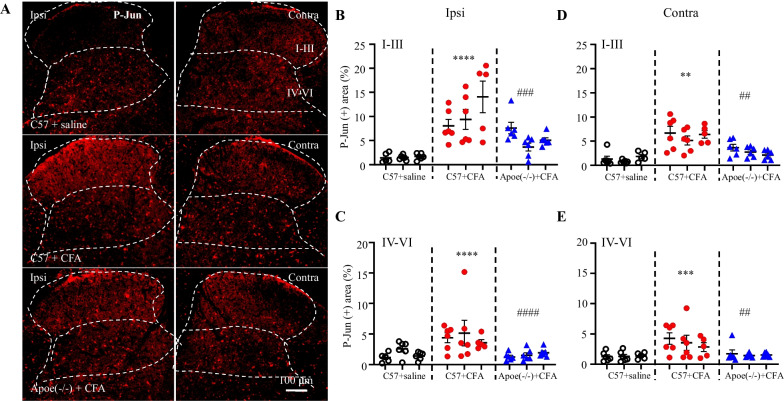
*Apoe* knockout suppresses the complete Freund’s adjuvant (CFA)-induced upregulation of p-Jun in the spinal dorsal horn. Representative images (**A**) and quantification of p-Jun-positive signals in the superficial dorsal horn (Layers I–III; **B**, **D**) and deep dorsal horn (Layers IV–VI; **C**, **E**) on the ipsilateral (Ipsi) side (**B**, **C**) and the contralateral (Contra) sides (**D**, **E**). N = 3 mice/group (5–6 sections/mouse). **B** ^****^p < 0.0001 compared with physiological saline-treated C57BL6 (C57) mice; ^###^*p* = 0.0002 compared with CFA-treated C57 mice. **C** ^**^p = 0.0013 compared with saline-treated C57 mice; ^###^*p* = 0.0006 compared with CFA-treated C57 mice. **D** ^****^p < 0.0001 compared with saline-treated C57 mice; ^####^*p* < 0.0001 compared with CFA-treated C57 mice. **E** ^***^p = 0.0004 compared with saline-treated C57 mice; ^##^*p* = 0.0014 compared with CFA-treated C57 mice. Two-way analysis of variance, followed by Tukey’s *post-hoc* test

**Fig. 6:**
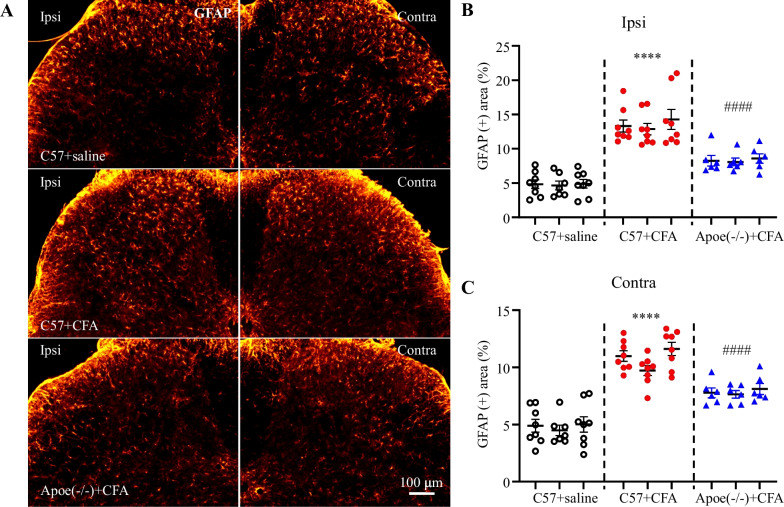
*Apoe* knockout suppresses GFAP expression in the spinal dorsal horn of the complete Freund’s adjuvant (CFA)-induced inflammatory pain mouse model. Representative images (**A**) and quantification (**B**, **C**) of GFAP-positive signals in the superficial dorsal horn on the ipsilateral (Ipsi) (**B**) and contralateral (Contra) sides (**C**). N = 3 mice/group (6–10 sections/mouse). ^****^p < 0.0001 compared with physiological saline-treated C57BL6 (C57) mice, ^####^*p* < 0.0001 compared with CFA-treated C57 mice. Two-way analysis of variance, followed by Tukey’s *post-hoc* test

Next, the regulatory effect of Apoe on astrocyte activation in the spinal dorsal horn of the CFA-induced inflammatory pain mouse model was examined. The p-Jun-positive and GFAP-positive areas were upregulated in the ipsilateral spinal dorsal horn of CFA-treated C57 mice (Figs. 5 and 6). However, the CFA-induced upregulation of p-Jun-positive and GFAP-positive areas in the ipsilateral and contralateral spinal dorsal horns was mitigated in *Apoe*^*−/−*^ mice (Figs. 5 and 6). These findings suggest that the antinociceptive effect of Apoe deficiency can be attributed to its inhibitory effects on astrocyte activation under CFA-induced inflammatory pain conditions.

### Intrathecal injection of *Apoe* ASO alleviates CFA-induced inflammatory pain

**Fig. 7:**
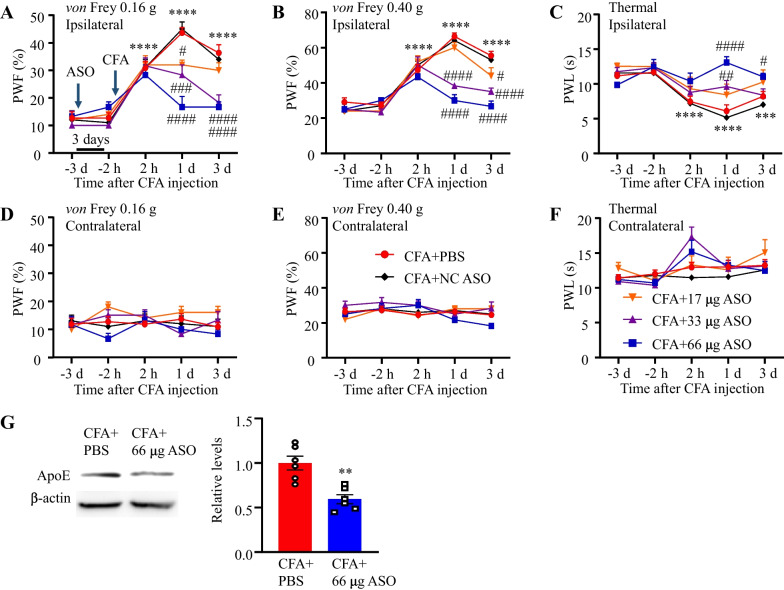
*Apoe* knockdown via intrathecal administration of *Apoe* antisense oligonucleotide (ASO) suppresses complete Freund’s adjuvant (CFA)-induced pain hypersensitivity. *Apoe* ASO was intrathecally administered 3 days and 1 h before CFA treatment. The behavioral tests were performed before *Apoe* ASO treatment and at 2 h and on days 1 and 3 post-CFA treatment. **A–F** The effect of *Apoe* ASO on CFA-induced pain hypersensitivity to 0.16-g *von* Frey filament (**A**, **D**), 0.4-g *von* Frey filament (**B**, **E**), and thermal stimuli (**C**, **F**) on the ipsilateral (**A**–**C**) and contralateral sides (**D**–**F**) after CFA treatment. N = 5–11 mice/group. ^****^p < 0.0001 and ^***^p = 0.0006 compared with phosphate-buffered saline (PBS) and CFA-treated mice at 2 h post-treatment; ^#^*p* < 0.05, ^##^*p* < 0.01, ^###^*p* < 0.001, ^####^*p* < 0.0001 compared with CFA and PBS-treated mice. Two-way analysis of variance, followed by Tukey’s *post-hoc* test. **G** The effect of *Apoe* ASO on Apoe expression in the spinal dorsal horn. N = 6 mice/group. ^**^p = 0.0013 compared with CFA and PBS-treated mice (two-tailed unpaired Student’s *t*-test)

Previously, *Apoe* ASO was reported to downregulate Apoe expression in the DRG [17]. The microinjection of *Apoe* ASO into the DRG or the intrathecal injection of *Apoe* ASO exerted antinociceptive effects on spinal nerve ligation-induced neuropathic pain [17]. This study examined the effect of *Apoe* ASO on CFA-induced inflammatory pain. ASO was intrathecally administered three days before CFA injection and the effect of ASO on pain hypersensitivity in CFA-treated mice was examined. CFA induced apparent mechanical allodynia and heat hyperalgesia on the ipsilateral paws at all tested time points after CFA injection (Fig. 7A–C), except for day 3 post-CFA treatment (partial recovery of heat hyperalgesia was observed) (Fig. 7C). The intrathecal injection of *Apoe* ASO dose-dependently attenuated CFA-induced mechanical allodynia (Fig. 7A, B) and heat hyperalgesia on days 1 and 3, but not at 2 h, post-CFA injection (Fig. 7C) on the ipsilateral side of ASO-treated mice. Treatment with 66 µg ASO produced the maximal effect on CFA-induced pain hypersensitivity (Fig. 7A–C). The intrathecal injection of ASO or vehicle did not affect the basal paw withdrawal responses on the contralateral side relative to the baseline level (Fig. 7D–F). Finally, the spinal tissues were harvested after behavioral tests and subjected to western blotting to analyze Apoe expression. *Apoe* ASO downregulated Apoe expression by 40% (Fig. 7G). These findings suggest that the intrathecal treatment of *Apoe* ASO exerts therapeutic effects on chronic inflammatory pain.

## Discussion

In this study, Apoe was upregulated in the spinal astrocytes of the CFA-induced inflammatory pain mouse model. Compared to the C57 mice, *Apoe*^*−/−*^ mice exhibited decreased pain hypersensitivity and paw edema inresponse to CFA injection. Consistently, the spinal dorsal horn Fos immunoreactivity, which serves as an indicator of neuronal activation [23, 24], in *Apoe*^*−/−*^ mice was less responsive when compared with that in C57 mice after CFA treatment. RNA sequencing and immunostaining analysis revealed spinal cellular changes associated to lipid metabolism and the glial activation in CFA-treated *Apoe*^*−/−*^ mice which may underlie the anti-nociceptive effect of Apoe deficiency. Additionally, treatment with *Apoe* ASO, which downregulates Apoe expression, decreased pain hypersensitivity in CFA-treated mice. Pain alleviation was not observed in *Apoe*^*−/−*^or *Apoe* ASO-treated mice at the early stage (2 h) of inflammation. This study demonstrated the crucial role of Apoe in persistent inflammatory pain.

Apoe along with high-density lipoproteins mediates lipid transport and cholesterol homeostasis in the CNS [25]. APOE is primarily expressed in astrocytes in both the brain and the spinal cord, as well as in the glia surrounding the sensory neurons in DRGs, and the non-myelinating Schwann cells of peripheral nerves [9, 17, 26]. The activation of microglia and astrocytes in the spinal cord is considered a key mechanism underlying the genesis of chronic pain [27]. This could explain the role of spinal astrocytic Apoe in the development of chronic pain. This study demonstrates that Apoe deficiency has antinociceptive effects during the late stages of inflammatory pain. Our previous study has demonstrated that Apoe in DRG glial cells is involved in the development of neuropathic pain in mice [17]. Additionally, as the essential role of Schwann cells in the development of neuropathic pain [28], Apoe in non-myelinated Schwann cells may mediate neuropathic pain. It requires further investigation. We also note that Apoe knockout has not been shown to have an effect on the basal nociceptive response, or on the acute phase of inflammatory pain. Moreover, we previously observed that the peripheral or intrathecal application of Apoe did not result in any nociceptive responses (licking, paw flicking, and paw withdrawing) [17]. This may be explained that acute pain was produced majorly associated with acute inflammation induced by inflammatory mediators (such as bradykinin, prostaglandins, H^+^, ATP, and nerve growth factor), pro-inflammatory cytokines, and chemokines released locally from neutrophils, macrophage, T cells, and mast cells [27].

RNA sequencing data revealed that *Apoe* knockout in the spinal dorsal horn exerts regulatory effects on synapse pruning, cholesterol metabolic processes, immune response, and activation of glial cells and T cells. Immunofluorescence analysis demonstrated that *Apoe* knockout suppresses astrocyte activation under CFA-induced inflammatory pain conditions. These data indicate the mechanism through which spinal astrocytic Apoe modulates pain processing under persistent inflammatory pain conditions. Astrocytes are the major source of lipids, providing nutritional support for the brain neurons [5, 6]. The lipid droplets in astrocytes play an essential physiological and protective role in the CNS [6]. Astrocytes release APOE that transports lipids and couples metabolism between astrocytes and neurons [6, 10]. Under pathological conditions, such as AD, acute stroke, and epilepsy, astrocytes can take up lipids from hyperactive neurons to exert protective effects against lipid toxicity, resulting in excessive astrocytic lipid accumulation and lipid metabolism reprogramming [29, 30]. The newly discovered subtype of reactive astrocytes, which are rich in lipids and exhibit the neurotoxic phenotype, induces cell death via saturated lipids contained in APOE lipoparticles to mediate astrocyte-induced toxicity [30, 31]. Astrocytes can take up toxic fatty acids secreted from hyperactive neurons in the form of APOE lipid-containing particles, which are stored in lipid droplets and metabolized in the mitochondria through β-oxidation [10, 29]. Thus, we hypothesize that Apoe upregulation in astrocytes of the spinal cord after CFA treatment indicates a reactive astrocyte with a neurotoxic phenotype. RNA sequencing data of spinal cord tissues demonstrated that *Apoe* knockout downregulated the expression of several genes involved in cholesterol biosynthesis, including *Cyp51*, *Hmgcs1*, and *Idi1*. Additionally, *Apoe* knockout upregulated *Abca1*, which encodes an extracellular phospholipid translocase, under inflammatory pain conditions. Thus, *Apoe* knockout in the spinal cord may downregulate cholesterol synthesis in astrocytes, preventing cholesterol and lipid accumulation in astrocytes, inhibiting the activation of astrocytes (indicated by expression of GFAP and p-Jun), and alleviating CFA-induced inflammatory pain.

Previous studies have reported that Apoe is mainly expressed in astrocytes it also has very low expression in microglia, as demonstrated in several studies [15, 17, 26]. In our RNA sequencing data, we noticed an increase in *cx3cr1* transcript levels in *Apoe*(-/-) mice compared with C57 mice. The *cx3cr1* gene encodes CX3CR1, which is primarily expressed in microglia and macrophages and has been implicated in neuropathic pain by affecting microglia-neuron crosstalk in the spinal dorsal horn [21, 32, 33]. *Apoe* is the top upregulated gene in spinal cord microglia at chronic time points in a peripheral nerve injury-induced neuropathic pain mouse model [15]. Additionally, *Apoe* was a hub gene in both macrophages and microglia in the subacute and chronic phases of spinal cord injury [34]. In mice, astrocytes with *Apoe* knockout exhibited upregulated matrisome pathway, chemotaxis, and inflammation. Meanwhile, *Apoe* knockout microglia exhibited upregulated cholesterol biosynthesis and other lipid pathways, predicting upregulated lipid synthesis [8]. Cholesterol metabolism in spinal microglia is associated with chemotherapy-induced neuropathic pain [35]. The long-lasting therapeutic effect of apolipoprotein A-I binding protein on neuropathic pain via cholesterol depletion was associated with anti-inflammatory effects and cholesterol metabolism reprogramming and decreased accumulation of lipid droplets in microglia [35]. Thus, APOE modulates the function of microglia in the spinal cord and is involved in CFA-induced inflammatory pain processing.

APOE mediates the transfer of cholesterol from astrocytes to neurons to form membranes, synaptic vesicles, and clusters of postsynaptic receptors [12, 25, 36]. Previous studies have demonstrated that the brain of *APOE4* allele carrier mice exhibits decreased dendritic length, spinal density, variable synaptic transmission, and long-term potentiation patterns, as well as the downregulation of synaptic proteins, such as Syp and Stx1a [36–38]. Furthermore, *APOE4* mice exhibited increased synaptic protein loss after lipopolysaccharide injection as evidenced by the downregulation of PSD-95, drebin, and synaptophysin [39]. Ibuprofen, a well-known nonsteroidal anti-inflammatory drug, exerts antinociceptive effects and can counteract the inhibitory effect of the *APOE4* genotype on dendritic spine density [37]. In this study, the less neuronal activity of spinal nociceptive neurons occurred in* Apoe*^*−/−*^ mice after CFA injection. Several DEGs associated with synapse pruning were identified in CFA-treated *Apoe*^*−/−*^mice. Thus, we hypothesized that *Apoe* knockout impairs the transport of cholesterol and lipids to the neurons. Consequently, the generation of new membranes in neurons, which is necessary for axon extension and dendritic spine production, is downregulated, contributing to pain alleviation in *Apoe*^*−/−*^ mice.

Humans have three *APOE* alleles/variants (*APOE2*, *APOE3*, and *APOE4*) [40]. The *APOE4* allele is the most important genetic risk factor for developing AD in humans [14, 41]. Among men, *APOE4* allele carriers have a decreased risk of developing chronic pain, whereas *APOE2* allele carriers are at an increased risk of developing chronic pain [15]. However, the *APOE2* allele is not correlated with chronic pain in women [15]. Furthermore, the *APOE4* allele is a protective factor against migraines and stress, whereas the *APOE2* allele is a risk factor [42]. Human APOE isoforms differ at amino acid positions 112 and 158 (cysteine at positions 112 and 158 in *APOE2*, a cysteine at position 112 and an arginine at 158 in *APOE3*, and arginine at positions 112 and 158 in *APOE4*) [43]. APOE4 is the risk factor for AD due to its toxic effect on lipid metabolism in microglia and astrocytes [8, 10]. Further studies are needed to understand the distinct roles of human APOE variants in different pathological conditions. In contrast to humans, mice harbor only a single *Apoe* allele [5]. The “target replacement” models of human APOE variants have been developed by replacing the *Apoe* allele of mice with one of the human *APOE* isoforms [44, 45]. These APOE target replacement mouse models can be used to study the differential role of human APOE isoforms in chronic pain.

## Conclusions

**Fig. 8:**
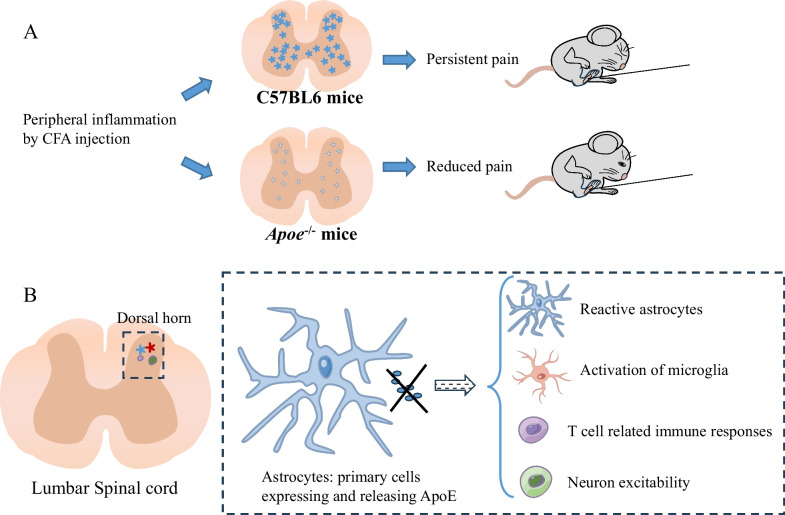
Schematic illustration of the role of Apoe in inflammatory pain. **A** The reduced pain in *Apoe*^*−/−*^ mice after complete Freund's adjuvant (CFA) injection. **B** *Apoe* deficiency exerts therapeutic effects on inflammatory pain by regulating various cellular functions

This study demonstrated the critical role of spinal Apoe in the maintenance of inflammatory pain. *Apoe* knockout exerts regulatory effects on the function of astrocytes, microglial cells, and neurons, alleviating inflammatory pain (Fig. 8). *Apoe* knockout may downregulate cholesterol synthesis in astrocytes, preventing the accumulation of cholesterol and lipids in astrocytes and consequently suppressing astrocyte activation. Thus, APOE can be a potential target for developing anti-nociception and anti-inflammation strategies.

### Supplementary Information

**Additional file 1**. RNA sequencing revealed 63 differentially expressed genes between *Apoe-/-* mice and C57BL6 mice in response to complete Freund's adjuvant (CFA) injection.

**Additional file 2**. The gene ontology analysis of 63 differentially expressed genes in biological processes.

**Additional file 3**. The gene ontology analysis of 63 differentially expressed genes in molecular functions.

**Additional file 4**. The kyoto encyclopedia of genes and genomes pathway analysis of 63 differentially expressed genes.

## Data Availability

The data that support the findings of this study are available from the corresponding author upon reasonable request. The RNA sequencing data are deposited in the BIG Sub repository (https://ngdc.cncb.ac.cn/gsa/), accession number CRA011487.

## Abbreviations

Apoe: Apolipoprotein E
ASO: Antisense oligonucleotide
CFA: Complete Freund's adjuvant
DEGs: Differentially expressed genes
DRG: Dorsal root ganglion
GFAP: Glial fibrillary acidic protein
GO: Gene ontology
JNK1: C-Jun N-terminal kinase 1
KEGG: Kyoto encyclopedia of genes and genomes
PBS: Phosphate-buffered saline
PMSF: Phenylmethanesulfonyl fluoride
PPI: Protein-protein interaction
PWF: Paw withdrawal frequency
PWL: Paw withdrawal latency
qPCR: Quantitative polymerase chain reaction
RIPA: Radio immunoprecipitation assay lysis
TBST: Tris buffered saline with tween 20

## References

[1] Cohen SP, Vase L, Hooten WM (2021). Chronic pain: an update on burden, best practices, and new advances. Lancet.

[2] Ji RR, Chamessian A, Zhang YQ (2016). Pain regulation by non-neuronal cells and inflammation. Science.

[3] Khakh BS, Deneen B (2019). The emerging nature of astrocyte diversity. Annu Rev Neurosci.

[4] Arranz AM, De Strooper B (2019). The role of astroglia in Alzheimer's disease: pathophysiology and clinical implications. Lancet Neurol.

[5] Mahley RW (2016). Central nervous system lipoproteins ApoE and regulation of cholesterol metabolism. Arterioscler Thromb Vasc Biol.

[6] Chen ZL, Yuan ZQ, Yang SC, Zhu YF, Xue MQ, Zhang J, Leng LG (2023). Brain energy metabolism: astrocytes in neurodegenerative diseases. CNS Neurosci Ther.

[7] Xiong XY, Tang Y, Yang QW (2022). Metabolic changes favor the activity and heterogeneity of reactive astrocytes. Trends Endocrinol Metab.

[8] Tcw J, Qian L, Pipalia NH, Chao MJ, Liang SA, Shi Y, Jain BR, Bertelsen SE, Kapoor M, Marcora E (2022). Cholesterol and matrisome pathways dysregulated in astrocytes and microglia. Cell.

[9] Lane-Donovan C, Herz J (2017). ApoE, ApoE receptors, and the synapse in Alzheimer's disease. Trends Endocrinol Metab.

[10] Lindner K, Beckenbauer K, van Ek LC, Titeca K, de Leeuw SM, Awwad K, Hanke F, Korepanova AV, Rybin V, van der Kam EL (2022). Isoform- and cell-state-specific lipidation of ApoE in astrocytes. Cell Rep.

[11] Zhang Y, Sloan SA, Clarke LE, Caneda C, Plaza CA, Blumenthal PD, Vogel H, Steinberg GK, Edwards MSB, Li G (2016). Purification and characterization of progenitor and mature human astrocytes reveals transcriptional and functional differences with mouse. Neuron.

[12] Dhillon H, Singh S (2018). Role of apolipoprotein E in the tangled mystery of pain. Med Hypotheses.

[13] Fullerton SM, Strittmatter WJ, Matthew WD (1998). Peripheral sensory nerve defects in apolipoprotein E knockout mice. Exp Neurol.

[14] Saunders AM, Strittmatter WJ, Schmechel D, George-Hyslop PH, Pericak-Vance MA, Joo SH, Rosi BL, Gusella JF, Crapper-MacLachlan DR, Alberts MJ (1993). Association of apolipoprotein E allele epsilon 4 with late-onset familial and sporadic Alzheimer's disease. Neurology.

[15] Tansley S, Uttam S, Urena Guzman A, Yaqubi M, Pacis A, Parisien M, Deamond H, Wong C, Rabau O, Brown N (2022). Single-cell RNA sequencing reveals time- and sex-specific responses of mouse spinal cord microglia to peripheral nerve injury and links ApoE to chronic pain. Nat Commun.

[16] VanderPutten DM, Cameron BM, Merril CR (1993). Increased apolipoprotein-E concentrations in individuals suffering chronic low back syndrome identified by two-dimensional gel electrophoresis. Appl Theor Electrophor.

[17] Liu S, Yang S, Zhou X, Zhu X, Xu L, Li X, Gao Z, Sun T, Wei J, Tian L (2023). Nerve injury-induced upregulation of apolipoprotein E in dorsal root ganglion participates in neuropathic pain in male mice. Neuropharmacology.

[18] Xu Q, Bernardo A, Walker D, Kanegawa T, Mahley RW, Huang Y (2006). Profile and regulation of apolipoprotein E (ApoE) expression in the CNS in mice with targeting of green fluorescent protein gene to the ApoE locus. J Neurosci.

[19] Zhao JY, Liang L, Gu X, Li Z, Wu S, Sun L, Atianjoh FE, Feng J, Mo K, Jia S (2017). DNA methyltransferase DNMT3a contributes to neuropathic pain by repressing Kcna2 in primary afferent neurons. Nat Commun.

[20] Liang L, Wei J, Tian L, Padma Nagendra BV, Gao F, Zhang J, Xu L, Wang H, Huo FQ (2020). Paclitaxel induces sex-biased behavioral deficits and changes in gene expression in mouse prefrontal cortex. Neuroscience.

[21] Zhang ZJ, Jiang BC, Gao YJ (2017). Chemokines in neuron-glial cell interaction and pathogenesis of neuropathic pain. Cell Mol Life Sci.

[22] Gao YJ, Xu ZZ, Liu YC, Wen YR, Decosterd I, Ji RR (2010). The c-Jun N-terminal kinase 1 (JNK1) in spinal astrocytes is required for the maintenance of bilateral mechanical allodynia under a persistent inflammatory pain condition. Pain.

[23] Herrera DG, Robertson HA (1996). Activation of c-fos in the brain. Prog Neurobiol.

[24] Vincent K, Wang SF, Laferriere A, Kumar N, Coderre TJ (2017). Spinal intracellular metabotropic glutamate receptor 5 (mGluR5) contributes to pain and c-fos expression in a rat model of inflammatory pain. Pain.

[25] Mahley RW (2016). Central nervous system lipoproteins: ApoE and regulation of cholesterol metabolism. Arterioscler Thromb Vasc Biol.

[26] Boyles JK, Pitas RE, Wilson E, Mahley RW, Taylor JM (1985). Apolipoprotein E associated with astrocytic glia of the central nervous system and with nonmyelinating glia of the peripheral nervous system. J Clin Invest.

[27] Ji RR, Xu ZZ, Gao YJ (2014). Emerging targets in neuroinflammation-driven chronic pain. Nat Rev Drug Discov.

[28] Campana WM (2007). Schwann cells: activated peripheral glia and their role in neuropathic pain. Brain Behav Immun.

[29] Ioannou MS, Jackson J, Sheu SH, Chang CL, Weigel AV, Liu H, Pasolli HA, Xu CS, Pang S, Matthies D (2019). Neuron-astrocyte metabolic coupling protects against activity-induced fatty acid toxicity. Cell.

[30] Guttenplan KA, Weigel MK, Prakash P, Wijewardhane PR, Hasel P, Rufen-Blanchette U, Münch AE, Blum JA, Fine J, Neal MC (2021). Neurotoxic reactive astrocytes induce cell death via saturated lipids. Nature.

[31] Chen ZP, Wang SJ, Zhao XS, Fang W, Wang ZG, Ye HJ, Wang MJ, Ke L, Huang TF, Lv P (2023). Lipid-accumulated reactive astrocytes promote disease progression in epilepsy. Nat Neurosci.

[32] Yi MH, Liu YU, Liu K, Chen T, Bosco DB, Zheng J, Xie M, Zhou L, Qu W, Wu LJ (2021). Chemogenetic manipulation of microglia inhibits neuroinflammation and neuropathic pain in mice. Brain Behav Immun.

[33] Clark AK, Yip PK, Grist J, Gentry C, Staniland AA, Marchand F, Dehvari M, Wotherspoon G, Winter J, Ullah J (2007). Inhibition of spinal microglial cathepsin S for the reversal of neuropathic pain. Proc Natl Acad Sci USA.

[34] Yao XQ, Chen JY, Yu ZH, Huang ZC, Hamel R, Zeng YQ, Huang ZP, Tu KW, Liu JH, Lu YM (2022). Bioinformatics analysis identified apolipoprotein E as a hub gene regulating neuroinflammation in macrophages and microglia following spinal cord injury. Front Immunol.

[35] Navia-Pelaez JM, Choi SH, Capettini LDA, Xia YN, Gonen A, Agatisa-Boyle C, Delay L, dos Santos GG, Catroli GF, Kim J (2021). Normalization of cholesterol metabolism in spinal microglia alleviates neuropathic pain. J Exp Med.

[36] Love S, Siew LK, Dawbarn D, Wilcock GK, Ben-Shlomo Y, Allen SJ (2006). Premorbid effects of APOE on synaptic proteins in human temporal neocortex. Neurobiol Aging.

[37] DiBattista AM, Dumanis SB, Newman J, Rebeck GW (2016). Identification and modification of amyloid-independent phenotypes of APOE4 mice. Exp Neurol.

[38] Sun GZ, He YC, Ma XK, Li ST, Chen DJ, Gao M, Qiu SF, Yin JX, Shi J, Wu J (2017). Hippocampal synaptic and neural network deficits in young mice carrying the human APOE4 gene. CNS Neurosci Ther.

[39] Zhu Y, Nwabuisi-Heath E, Dumanis SB, Tai LM, Yu C, Rebeck GW, LaDu MJ (2012). APOE genotype alters glial activation and loss of synaptic markers in mice. Glia.

[40] Toro CA, Das DK, Cai D, Cardozo CP (2019). Elucidating the role of apolipoprotein E isoforms in spinal cord injury-associated neuropathology. J Neurotrauma.

[41] Strittmatter WJ, Saunders AM, Schmechel D, Pericak-Vance M, Enghild J, Salvesen GS, Roses AD (1993). Apolipoprotein E: high-avidity binding to beta-amyloid and increased frequency of type 4 allele in late-onset familial Alzheimer disease. Proc Natl Acad Sci U S A.

[42] Gupta R, Kumar V, Luthra K, Banerjee B, Bhatia MS (2009). Polymorphism in apolipoprotein E among migraineurs and tension-type headache subjects. J Headache Pain.

[43] Corder EH, Saunders AM, Strittmatter WJ, Schmechel DE, Gaskell PC, Small GW, Roses AD, Haines JL, Pericakvance MA (1993). Gene dose of apolipoprotein-E type-4 allele and the risk of alzheimers-disease in late-onset families. Science.

[44] Castellano JM, Kim J, Stewart FR, Jiang H, DeMattos RB, Patterson BW, Fagan AM, Morris JC, Mawuenyega KG, Cruchaga C (2011). Human apoE isoforms differentially regulate brain amyloid-β peptide clearance. Sci Transl Med.

[45] Bales KR, Liu F, Wu S, Lin SZ, Koger D, DeLong C, Hansen JC, Sullivan PM, Paul SM (2009). Human isoform-dependent effects on brain β-amyloid levels in PDAPP transgenic mice. J Neurosci.

